# Supplementary material for: Statins Show Anti-Atherosclerotic Effects by Improving Endothelial Cell Function in a Kawasaki Disease-like Vasculitis Mouse Model
Source: Int J Mol Sci. 2022 Dec 17;23(24):16108. doi: 10.3390/ijms232416108 (PMC9780952; doi:10.3390/ijms232416108)
Supplement: Supplementary file 1 [file ijms-23-16108-s001.zip › ijms-2062406-supplementary.pdf]

Supplementary data S1

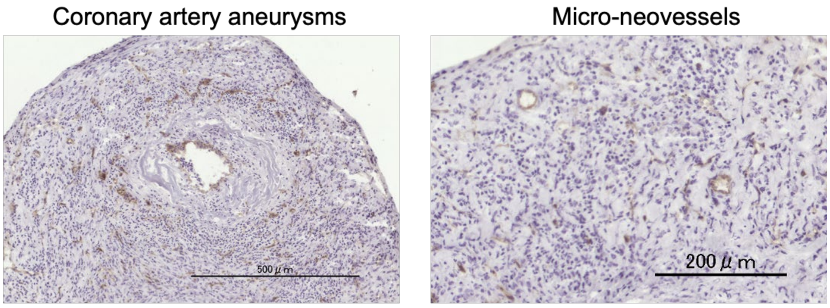

In the endothelial epithelium of the aorta, eNOS was suppressed by CAWS treatment. On the other hand, inflammation was observed at the aortic root in all CAWS-treated individuals, and eNOS expression was strong in the endovascular epithelium of the coronary artery aneurysms and the neovascular micro-neovessels of the adventitia.
